# Health-related quality-of-life in adolescent idiopathic scoliosis patients 25 years after treatment

**DOI:** 10.1186/s13013-015-0045-8

**Published:** 2015-07-16

**Authors:** Ane Simony, Emil Jesper Hansen, Leah Y. Carreon, Steen Bach Christensen, Mikkel Osterheden Andersen

**Affiliations:** Sector for Spine Surgery & Research, Middelfart Hospital Denmark, Ostre Hougvej 55, 5500 Middelfart, DK Denmark

**Keywords:** Long-term outcome, Health related quality of life, Adolescent idiopathic scoliosis, Harrington-DTT, Boston braces

## Abstract

**Background:**

Since 1962 to the mid eighties the Harrington Rod instrumentation was the Golden standard for surgical treatment of Adolescent Idiopathic Scoliosis (AIS). The Boston braces were introduced in the 1970´s and are still used as a conservative treatment, for curves less than 40°. Very few long-term studies exists, focusing on the health related quality of life. The purpose of this study was to evaluate the long-term health related outcome, in a cohort of AIS patients, treated 25 years ago.

**Method:**

219 consecutive patients treated with Boston brace (Brace) or posterior spinal fusion (PSF) using Harrington- DDT instrumentation between 1983 and 1990 at Rigshospitalet Copenhagen, were invited to participate in a long-term evaluation study. A validated Danish version of the Scoliosis Research Society 22R (SRS22R) and Short Form-36 (SF36v1) were administrated to the patients two weeks before the clinical and radiological examination.

**Results:**

159 (72,6 %) patients participated in the clinical follow up and questionnaires, 11 patients participated only in the questionnaires, 8 emigrated, 4 were excluded due to progressive neurological disease and 2 were deceased. The total follow up was 170 patients (83 %), and the average follow up was 24.5 years (22–30 years).

SRS22R domain scores were within the range described as normal for the general population with no statistical difference between the groups except in the Satisfaction domain, where the PSF group had better scores than the braced group.

The SF36 PCS and MCS scores in both AIS cohorts were similar to the scores for the general population.

**Conclusion:**

HRQOLs, as measured by the SRS22R and SF-36, of adult AIS patients treated with Boston brace or PSF during adolescence were similar to the general population. No clinical progression of the deformity has been detected during the 25-year follow up period. The PSF group had a small but statistically significant higher score in the Satisfaction domain compared to the braced group.

**Trial registration:**

S-20110025 Regional Committees on Health Research Ethics for Southern Denmark.

## Introduction

Scoliosis is a three dimensional deformity of the spine that affects 1-2 % of the population, mostly females. The most common subgroup is Adolescent Idiopathic Scoliosis (AIS) [1–4]. AIS is often diagnosed during the rapid growth period of the spine, usually from age 11–14 years [3]. Untreated AIS can progress to severe deformation of the spine, with severe pulmonary complication, back pain and reduced health related quality of life [5–7]. Brace treatment and surgical correction are the most common treatment modalities, and both treatments are well documented to correct the spinal deformity. Both these treatments are performed prophylactically, that is to prevent disease progression and the sequelae that are foreseen to occur two to three decades after the initiation of treatment. However, there are a very few studies with a follow-up longer than 20 years that have been published [8–12]. The purpose of the study was to evaluate the long-term health related outcome after treatment of AIS patients with either a Boston brace or Harrington-DTT instrumentation at a minimum of 20 years follow up.

## Methods

Two hundred nineteen consecutive patients, treated with either a module Boston Braces or posterior spinal fusion with Harrington-DTT instrumentation for AIS at University Hospital Rigshospitalet, Copenhagen from 1983–1990 were invited to participate. Boston brace treatment was initiated in patients with progression of the deformity, curves larger than 30°, and had remaining growth potential assessed by Risser sign [13, 14] and onset of menarche. Patients were instructed to wear the brace for 23 h a day and were evaluated twice a year by a paediatric orthopaedic surgeon. Bracing was continued until the patient achieved full standing height, was two years post menarcheal or was Risser 5. Patients with curves larger than 50° at the time of referral, or with curve progression despite brace treatment were treated surgically with posterior spinal fusion and Harrington-DTT instrumentation. Surgical patients were placed in serial post-operative casts to ensure a solid spinal fusion. A 10 years follow-up from the same patient population, has previously been published by Andersen et al. in 2006 [15].

The patients were invited to participate in a clinical and radiological examination, including health related quality of life (HRQL) questionnaires. The original medical charts from first visit to termination of treatment angles were available including Cobb angel measurements, curve types and end vertebrae recorded at every visit. All radiographic evaluations were performed by the authors MA and SBC. The original radiographs were no longer available. After obtaining a written consent, patients were invited to a clinical examination and full-length 36-inch posteroanterior and lateral standing radiographs were taken. A Danish version of Short Form-36 (SF-36) [16], Scoliosis Research Society Instrument-22R (SRS-22R) [17] and EuroQOL-5D (EQ5D) [18] was also administered.

All statistical analyses were carried out using PASW V.17.0 (IMB, Somes, NY). Comparisons between the braced and the surgical group were performed using unpaired t-tests for normally distributed continuous variables, Wilcoxon signed rank test for continuous variables that were not normally distributed and Fisher’s exact test for categorical variables.

## Results

Of the 219 patients who met inclusion criteria, 159 (73 %) participated in the clinical and radiological follow up. Eleven (5 %) completed HRQOL surveys but did not consent to the clinical and radiological examination. Thirty-three patients did not respond to the follow up invitation, 8 emigrated and were not available for follow up, 4 were excluded due to progressive neurological disease and 2 were deceased.

The total follow up was 170 patients (83 %), 73 of whom were braced and 97 treated with surgery. The average age at follow up was 41.4 years (range, 34 – 47) in the braced group and 37.6 (range, 33–47) in the surgery group. The average length of follow up was 26.5 years (range, 22–31 years) in the braced group and 24.5 years (range, 22–30 years) in the surgery group (Table 1).

**Table 1:** Demographic characteristics of patients

	Boston brace	Harrington rod instrumentation	Total
N	73	97	170
Female	62	85	147
Age at end of treatment (years)	15.8 (14–19)	14.3 (12–18)	
Age at follow-up (years)	41.4 (34–47)	37.6 (33–47)	

Although all the SRS22-R domain scores were within the range described as normal for the general population [19], the scores were equal between the surgery group, compared to the braced group. Only in the Satisfaction with management domain, there was a statistical significance between the 2 groups (Table 2).

**Table 2:** Summary of outcome scores

Outcome Measure	Boston brace	Harrington rod instrumentation	*p*-value
Scoliosis Research Society – 22R	Mean (SD)	Mean (SD)	
Function	4.21 (0.82)	4.30 (0.83)	0.281
Pain	3.73 (0.95)	3.86 (0.97)	0.262
Self-Image	3.60 (0.94)	3.83 (0.82)	0.161
Mental	3.99 (0.74)	4.07 (0.77)	0.442
Satisfaction	3.46 (0.91)	4.33 (0.76)	0.000
Short Form - 36			
Physical Composite Summary	47.87 (10.26)	47.54 (11.20)	0.780
Mental Composite Summary	50.38 (11.32)	53.07 (9.24)	0.070
EuroQOL 5D			
Score	0.82 (0.15)	0.85 (0.16)	0.213

The SF36 PCS and MCS scores in both AIS cohorts were similar to the scores for the Danish age-matched general population, published by Bjoerner [20], with no statistically significant difference between the two groups. Similarly, there was no difference in the EQ-5D scores between the braced and surgery groups.

In the surgery group, 4 had revision surgery due to proximal implant failures and 3 due to broken rods before solid fusion was achieved; 3 patients had rod fractures within the first year after surgery, but the correction was maintained and there was no soft tissue irritation, so the rod was left in place; 3 patients complained of postoperative thoracic pain which resolved within the first postoperative year; and two patients developed pneumo thorax during resection of their rib deformity. There were no deep wound infections and no neurologic complications.

## Discussion

Few studies have been published on the long-term outcome in AIS patients, focusing on the health related quality of life. Although studies reporting the quality of life in patients treated surgically for scoliosis have been available, [4, 8–12, 21, 22] these studies were largely limited by small sample size and the inclusion of congenital and neuromuscular scoliosis patients;[21] by the lack of a generic measure of quality of life [8, 9], or by the lack of surgical treatment of patients in the cohort [4].

In the current study, HRQOL as measured by SF36 MCS and PCS in the AIS patients were similar to normal age-matched Danish normal data [20], regardless the treatment the patients had received. These findings are similar to the study by Danielsson [11] and Gotze [23], looking at long term health related quality of life measures in AIS patients treated with Harrington-DTT instrumentation. Danielsson [11] evaluated AIS patients treated with surgery with at least 20 year follow-up and reported that in their surgical cohort with a mean age of 39.7 years, the mean SF-36 PCS score was 50.9 and the mean SF-36 MCS score was 50.2. In a recent study looking at AIS patients who had Harrington-DTT instrumentation, patients at a mean age of 32.3 years had a mean score of 50.9 for both the SF-36 PCS and MCS [23]. Neither of these studies used the disease specific SRS-22R as an outcome measure.

Disease specific outcomes evaluated by the SRS 22R domain scores were similar to the normal age-matched scores [19] and showed no statistically significant difference between the braced and the surgery group, except in the Satisfaction with management scores. In an earlier study on the same cohort of patients, ten years after the end of treatment, Andersen reported that the daily 23 h brace treatment significantly decreased the patient’s daily activities, and that the brace treated patients experienced more back pain than the surgical treated patients [15]. Brace related questions revealed a negative impact on the patients Activity of Daily Living, participation in Sport, and some kept their brace treatment as a secret to friends. This negative impact of brace wear [24, 25] may help explain the difference in the Satisfaction with treatment domain, in these patients.

Spinal deformity correction is performed to prevent impaired pulmonary function and spine related disability later in life [6, 7]. Thus, longer term studies, when patients are in their fifth and sixth decade, are needed to determine whether these patients will have similar quality of life outcomes, pulmonary function and spine related problems as the general population.

## Conclusion

Health related quality of life, as measured by the SRS22R and SF-36, of adult AIS patients treated with Boston brace or surgery during adolescence were similar to the general population. No clinical progression of the deformity has been detected during the 25-year follow up period.
